# Feasibility insights into the application of *Paenibacillus pabuli* E1 in animal feed to eliminate non-starch polysaccharides

**DOI:** 10.3389/fmicb.2023.1205767

**Published:** 2023-08-07

**Authors:** Gen Li, Yue Yuan, Bowen Jin, Zhiqiang Zhang, Bilal Murtaza, Hong Zhao, Xiaoyu Li, Lili Wang, Yongping Xu

**Affiliations:** ^1^School of Bioengineering, Dalian University of Technology, Dalian, China; ^2^School of Biological Engineering, Dalian Polytechnic University, Dalian, China

**Keywords:** NSPs, DDGS, degradation, *Paenibacillus*, fermentation

## Abstract

The goal of the research was to find alternative protein sources for animal farming that are efficient and cost-effective. The researchers focused on distillers dried grains with solubles (DDGS), a co-product of bioethanol production that is rich in protein but limited in its use as a feed ingredient due to its high non-starch polysaccharides (NSPs) content, particularly for monogastric animals. The analysis of the *Paenibacillus pabuli* E1 genome revealed the presence of 372 genes related to Carbohydrate-Active enzymes (CAZymes), with 98 of them associated with NSPs degrading enzymes that target cellulose, hemicellulose, and pectin. Additionally, although lignin is not an NSP, two lignin-degrading enzymes were also examined because the presence of lignin alongside NSPs can hinder the catalytic effect of enzymes on NSPs. To confirm the catalytic ability of the degrading enzymes, an *in vitro* enzyme activity assay was conducted. The results demonstrated that the endoglucanase activity reached 5.37 U/mL, while beta-glucosidase activity was 4.60 U/mL. The filter paper experiments did not detect any reducing sugars. The xylanase and beta-xylosidase activities were measured at 11.05 and 4.16 U/mL, respectively. Furthermore, the pectate lyase and pectin lyase activities were found to be 8.19 and 2.43 U/mL, respectively. The activities of laccase and MnP were determined as 1.87 and 4.30 U/mL, respectively. The researchers also investigated the effect of *P. pabuli* E1 on the degradation of NSPs through the solid-state fermentation of DDGS. After 240 h of fermentation, the results showed degradation rates of 11.86% for hemicellulose, 11.53% for cellulose, and 8.78% for lignin. Moreover, the crude protein (CP) content of DDGS increased from 26.59% to 30.59%. In conclusion, this study demonstrated that *P. pabuli* E1 possesses various potential NSPs degrading enzymes that can effectively eliminate NSPs in feed. This process improves the quality and availability of the feed, which is important for animal farming as it seeks alternative protein sources to replace traditional nutrients.

## 1. Introduction

Pigs play a pivotal role as a primary source of meat and protein for human consumption (Casellas et al., 2013). The demand for pig feed is exceptionally high, accounting for over 20% of the total animal feed production (Mottet et al., 2017). With the current global maize production reaching 114.8 million tons (Wang et al., 2021), maize serves as the predominant raw material in swine feed formulation. However, the escalating demand for maize in the swine industry poses a significant challenge, as it competes directly with human food sources, thereby compromising long-term sustainability. Consequently, the development of nutritious and efficient swine feeds assumes strategic significance in addressing the pressing issue of global food shortages.

The United States and Brazil hold significant prominence as the foremost global producers of fuel ethanol. In the year 2021, the collective global production of bioethanol amounted to a substantial 27.22 billion gallons. Among the major contributors, the United States spearheaded the production with a noteworthy output of 15.01 billion gallons, closely followed by Brazil at 7.43 billion gallons. Remarkably, the combined efforts of the United States and Brazil accounted for an impressive 82.3% of the world’s total ethanol production (Filipe et al., 2023). Currently, corn ethanol production is predominant in the United States, while sugarcane ethanol production is predominant in Brazil. A significant by-product of sugarcane processing is bagasse (Sugarcane Bagasse SB), which contains approximately 2.1% to 2.9% crude protein, 79.4% to 88.3% neutral detergent fiber, 62.2% to 69.8% acid detergent fiber, 22.1% total lignin, 10.3% to 10.5% acid detergent lignin, 1.4% ash, and some extracts (Arntzen et al., 2021; de Lucas et al., 2021). Bagasse has low crude protein content and high cellulose and lignin content, making it suitable for ruminant feed. Ruminants have various microorganisms in their rumen that can digest and decompose cellulose and hemicellulose (de Almeida et al., 2018). On the other hand, distillers dried grains with solubles (DDGS), a co-product of corn fermentation, is high in protein and can serve as a substitute protein feed ingredient (Streams, 2006; Pahm et al., 2009). Corn is the primary grain used for ethanol production in the United States and China. Approximately 1.4 L of ethanol and 1 kilogram of DDGS are produced from every 3 kilograms of fermented corn (Mohammadi Shad et al., 2021). DDGS has been recognized as a valuable source of protein, energy, water-soluble vitamins, lutein, and linoleic acid (Lumpkins et al., 2005) and is utilized as an alternative ingredient in swine feed. The global annual output of DDGS exceeds 40 million tons, with China alone producing over 15 million tons. As a new high-quality protein resource, DDGS can help reduce the overall amount of feed required and partially alleviate the significant feed shortage (Abd El-Hack et al., 2018).

Non-starch polysaccharides (NSPs) are widely distributed anti-nutritional factors found in plant-derived feeds. Cereals, including corn, have high levels of NSPs, mainly composed of pentosan, glucan, and cellulose. Compared to corn, DDGS has varying crude protein content ranging from 26.7% to 32.9%, with concentrated levels of NSPs (Min et al., 2009). DDGS contains xylan ranging from 9.1% to 18.4% and cellulose ranging from 6.3% to 14.7%. Monogastric animals lack the enzymes necessary to digest cellulose and xylan. NSPs increase chyme viscosity and hinder the interaction between digestive enzymes and nutrients, thereby greatly affecting nutrient absorption and utilization (Xu et al., 2009). As anti-nutritional factors, NSPs disrupt the physiological activities of intestinal microorganisms, reduce animal production performance, and have a more significant impact on young animals (Pedersen et al., 2014; Swiatkiewicz et al., 2016). Cellulose, a linear polymer of D-glucose units linked by beta-1,4-glycosidic bonds, forms microfibril units through hydrogen bonding between cellulose chains. These microfibril units assemble to create cellulose fibers, enhancing the stability and resistance of the cell wall against degradation (Thapa et al., 2020). Endo-1,4-beta-D-glucanases hydrolyze beta-1,4 linkages randomly in both soluble and insoluble cellulose chains. Cellobiohydrolases (CBHs) release cellobiose from the reducing (CBH II) and non-reducing (CBH I) ends of cellulose chains. Beta-glucosidases liberate D-glucose. Hemicellulose consists of pentose sugars (beta-D-xylose, alpha-L-arabinose), hexose sugars (beta-D-mannose, beta-D-glucose, and alpha-D-galactose), and aldonic acid (alpha-D-glucuronic acid; Girio et al., 2010; Escamilla-Alvarado et al., 2017). Hemicellulases can be categorized into three types: endoenzymes that act within the interior of polysaccharides, exoenzymes that hydrolyze from either the reducing or non-reducing ends, and coenzymes that act on branched chains. Hemicellulases encompass various enzymes such as xylanases, mannanases, beta-glucanases, galactanases, ferulic acid esterases, acetyl esterases, and arabinofuranosidases. Lignin, a three-dimensional biopolymer, forms complex structures composed of random propanol groups. It acts as a barrier, preventing cellulolytic enzymes from accessing their substrates by physically obstructing enzyme-cellulose interaction and hindering contact with hemicelluloses, leading to non-productive enzyme adsorption (Meng et al., 2020). Lignin-degrading enzymes primarily include laccases, manganese peroxidases, and lignin peroxidases (Bugg and Rahmanpour, 2015).

Consequently, the elimination of NSPs in feed ingredients is necessary. *Paenibacillus* species are widely distributed in various environments, particularly in soil, where they play roles in detoxification through biological nitrogen fixation (Xie et al., 2014), phosphate dissolution (Xie et al., 2016), production of the plant hormone indole-3-acetic acid (IAA; Patten et al., 2013), and release of siderophores (Raza and Shen, 2010), which promote crop growth. Some bacteria, including *Paenibacillus* species, produce antimicrobial agents such as bacteriocins and antimicrobial peptides that can be used to control phytopathogenic microorganisms, reducing the need for chemical fungicides that may negatively impact the environment (Xie et al., 2016). Various *Paenibacillus* species found in the soil produce glucans, chitinases, cellulases, and proteases involved in the degradation of eukaryotic cell walls (Grady et al., 2016; Seo et al., 2016). However, there are limited reports on the systematic study of non-starch polysaccharide degradation by *Paenibacillus*. The target bacteria in this study belong to the *Paenibacillus* genus. The purpose of this study is to explore the degradation effect of the target bacteria on non-starch polysaccharides in DDGS and improve the feeding value of DDGS.

*Paenibacillus pabuli* E1 was isolated from surface soil and stored in our laboratory. It has been thoroughly characterized and its genome has been completely sequenced. Carbohydrate-active enzymes (CAZymes) are a group of enzymes that degrade, modify, or generate glycosidic bonds, enabling efficient carbohydrate utilization. The CAZy database categorizes CAZymes into four types: glycoside hydrolases (GHs), glycosyl transferases (GTs), polysaccharide lyases (PLs), and carbohydrate esterases (CEs). The database also includes carbohydrate-binding modules (CBMs; Cantarel et al., 2009). In this study, we report the annotation of carbohydrate-active enzymes (CAZymes) of *P. pabuli* E1. The NSPs-degrading genes in the *P. pabuli* E1 genome were identified, and the structural information of different NSPs-degrading enzymes was comprehensively analyzed. The identification results based on CAZymes provide insights into the degradation mechanism of NSPs in feed and contribute to the design and development of useful microorganisms and enzyme preparations for agriculture or feed. This study investigated the degradation ability of *P. pabuli* E1 on NSPs in DDGS and provides guidance for the fermentation application of *P. pabuli* E1 in eliminating NSPs in feed. The results offer a theoretical basis for the subsequent development of NSPs enzyme preparations.

## 2. Materials and methods

### 2.1. CAZyme annotation

The GenBank accession number for *P. pabuli* E1 is MT322455, and the strain preservation number is CGMCC NO.20517 (Li et al., 2021). All protein-encoding ORFs from *P. pabuli* E1 genomes were subjected to CAZy annotation using a two-step procedure of annotation and identification. BLASTp or Markov models were employed to confirm that the sequences belonged to the CAZyme family, and the information for each protein was collected (Bayer et al., 2008). The identification of CAZymes in *P. pabuli* E1 was performed using the HMMER (e-value < 1e^−15^, coverage > 0.35), and DIAMOND (e-value < 1e^−102^) tools in dbCAN (Tamaru et al., 2011). The results were analyzed to determine the presence of a secretory signal peptide or a transmembrane domain for each identified enzyme (Fabrice et al., 2002).

### 2.2. Growth condition

Luria-Bertani (LB) medium was composed of tryptone (20 g/L), yeast (10 g/L), and NaCl (20 g/L). Minimal mineral (MM) medium consisted of (NH_4_)_2_SO_4_ (1 g/L), NaH_2_PO_4_ (0.5 g/L), K_2_HPO_4_ (0.5 g/L), MgSO_4_ (0.2 g/L) and CaCl_2_ (0.1 g/L). The polysaccharide medium was MM solid medium supplemented with agar (15 g/L) and different polysaccharides (1 g/L) such as carboxyl methyl cellulose (CMC), filter paper, cellulose powder, xylan, and pectin. LB medium supplemented with the above-mentioned polysaccharides (0.5 g/L) in addition to cellulose powder was used to produce degradative enzymes. The ability to degrade lignin was also tested because it is closely related to the above polysaccharides. All media were autoclaved at 121°C for 20 min. Before each experiment, *P. pabuli* E1 was reactivated in a fresh LB liquid medium. Cultures were incubated at 37°C on a shaker at 160 rpm for 48 h. The presence of spores can hinder the production of degradative enzymes in *P. pabuli* E1 cultures for more than 48 h. Hence, a 48-h incubation time was adopted for our experiments.

### 2.3. Enzyme assay

*Paenibacillus pabuli* E1 was cultivated in the LB medium for 48 h. After 10 min centrifugation of liquid culture (6,000 ×*g* at 4°C), both supernatant and cell pellets were collected. The supernatant was used as crude enzyme source, while cell pellets were disrupted by ultrasonication (300 W, 2 s interval 4 s, 7 min) in an ice bath and then centrifuged at 4°C, 6,000 ×*g* for 10 min. The filter paper disintegration experiment assessed the supernatant and cell insoluble fraction separately. To initiate the experiment, 50 mL of the medium was added with 0.05 g of circular filter paper and incubated at 37°C and 160 rpm, with phosphate buffered saline (PBS) solution serving as a control. The endo-cellulase activity was determined using the 3,5-dinitrosalicylic acid (DNS) method, as described by Miller (1959). The assay was conducted by combining approximately 0.05 mL of crude enzyme with 0.3 mL of 1% carboxymethyl cellulose (CMC) that was solubilized in a 0.05 M PBS (pH 7.0). The mixture was then incubated at 40°C in a water bath for a duration of 30 min. Subsequently, 0.285 mL of DNS solution was added, and the reaction was stopped by boiling the mixture in a water bath for 10 min. The liberated sugars were quantified by measuring the absorbance at 540 nm. The xylanase and pectate lyase activities were quantified using the DNS method, with xylan and pectin employed as the respective substrates. Beta-glucosidase activity was estimated by spectroscopic measurement of *p*-nitrophenol (pNP) released from *p*-nitrophenyl-beta-glucopyranoside (pNPG). The reaction mixture contained 0.4 mL of 1 mM pNPG, 0.5 mL of 0.1 M PBS buffer (pH 7.0), and 0.1 mL of crude enzyme solution. The reaction mixture was incubated at 37°C for 30 min. The reaction was stopped by the addition of 1.0 mL of 0.5 M Na_2_CO_3_, centrifuged at 6,000 ×*g* for 5 min at 4°C, and measured the absorbance at 400 nm. Beta-xylosidase activity was similarly estimated under the same conditions by measurement of pNP released from *p*-nitrophenyl-beta-D-xylopyranoside (pNPX). Pectin lyase acted on the alpha-1,4 glycosidic bonds in pectin, generating unsaturated oligogalacturonic acid with unsaturated bonds between C4 and C5 at the reducing end, which exhibited a characteristic absorption peak at 235 nm. Laccase decomposed 2,2-azino-bis(3-ethylbenz-thiazoline-6-sulfonate; ABTS) to produce ABTS radicals, with a significantly higher absorption coefficient at 420 nm than the substrate ABTS. The molar extinction coefficient of ABTS was 36,000 L·mol^−1^·cm^−1^. In the presence of Mn^2+^, manganese peroxidase oxidized guaiacol to 4-o-methoxy phenol, which had an absorption peak at 465 nm. The activity of manganese peroxidase was determined by monitoring the change in absorbance at 465 nm. The molar extinction coefficient of guaiacol was 12,100 L·mol^−1^·cm^−1^. The MnP Enzyme Activity Detection Kit was obtained from Beijing Solarbio Science & Technology Co., Ltd., and other reagents were purchased from Sangon Biotech (Shanghai) Co., Ltd. The enzyme unit (U/mL) was calculated as the amount of enzyme required to release one μmol of reducing sugar or product per mL per minute.

### 2.4. Solid-state fermentation

DDGS was obtained from Weifang Yingxuan Industrial Co., LTD (Shandong, China). The inoculum concentration for DDGS fermentation was 10^7^–10^8^ CFU/mL. Sterile water was added to 50 g of feed, and the humidity was adjusted to 50% using a hygrometer. The mixture was then placed in a sterile fermentation bag with a one-way filter valve. Solid-state fermentation was conducted at 37°C for 240 h. The humidity of the solid-state ferment was monitored daily, and if reduced, it was supplemented with sterile water as needed. Samples were collected every 48 h for nutritional composition analysis. DDGS samples were dried at 105°C until a constant weight was achieved. Crude protein (CP) was determined by the Kjeldahl method (Belyea et al., 2004). The content of crude fiber (*CF*) was determined following the AOCS Ba 6a-5 standard method (Dey et al., 2021). The content of cellulose, hemicellulose, and Klason lignin was determined using a two-step acid treatment (Leite et al., 2016). Ash content was determined by incinerating samples in a muffle furnace at 450°C for 4 h.

### 2.5. Statistical analysis

Enzyme assays and solid-state fermentation experiments were performed in triplicate. Origin 2023 (Origin 2023 program, OriginLab) and SPSS v 25.0 were used for variance (ANOVA) and statistical comparisons, as well as for data visualization. Results were presented as means of more than three replicates.

## 3. Results

### 3.1. CAZy database annotation

**Figure 1:**
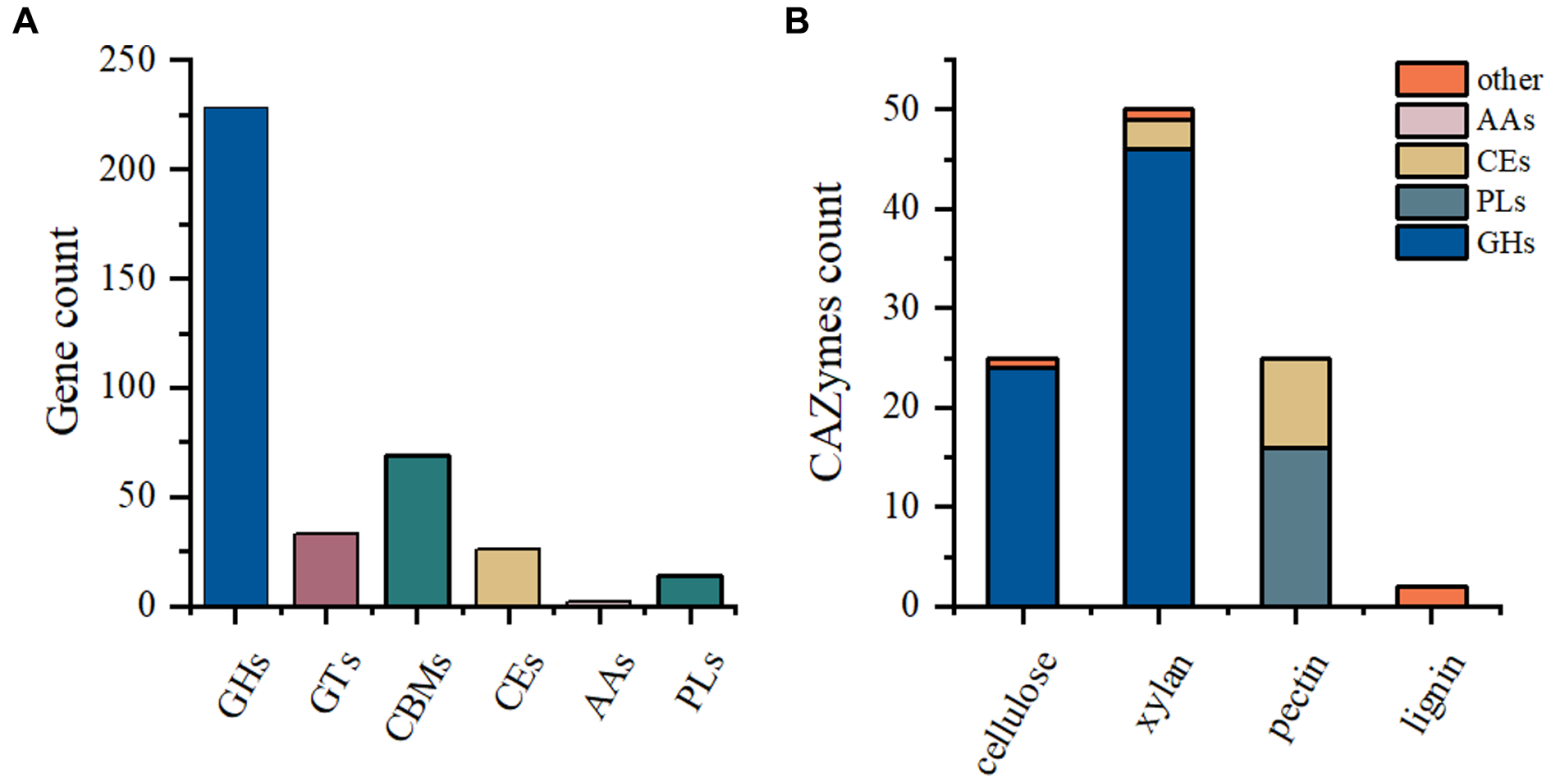
CAZyme annotation. **(A)** A total of 372 genes were assigned to CAZyme families. The GH family exhibited the highest abundance, followed by CBM, GT, CE, PL, and AA families, with 228, 69, 33, 26, 14, and 2 genes, respectively. **(B)** Among the GH families, 24 and 46 were associated with the degradation of cellulose and hemicellulose, respectively. The PL families contained 16 pectin-degrading enzymes. In the CE families, three and nine enzymes were related to the degradation of hemicellulose and pectin, respectively.

The *P. pabuli* E1 genome harbors a considerable number of carbohydrate-degrading enzymes. However, limited information on *Paenibacillus* species for feed is available in the existing database. Therefore, this study aimed to analyze the carbohydrate distribution in the *P. pabuli* E1 genome to provide fundamental data for the development and utilization of degrading enzymes. A total of 372 genes were assigned to CAZymes families. Among these, the GH family was the most predominant, followed by CBM, GT, CE, PL, and AA families, with gene counts of 228, 69, 33, 26, 14, and 2, respectively (Figure 1A). The GH family comprises a versatile group of enzymes involved in the hydrolysis of glycosidic bonds in carbohydrates. The abundance of GH family enzymes in *P. pabuli* E1 is advantageous for its growth and reproduction by facilitating the degradation of complex polysaccharides. Specifically, there are 24 GH families associated with cellulose degradation and 46 GH families related to hemicellulose degradation. Additionally, the PLs families encompass 16 pectin-degrading enzymes, while the CEs families comprise three and nine enzymes involved in hemicellulose and pectin degradation, respectively (Figure 1B).

#### 3.1.1. Cellulose degradation

**Figure 2:**
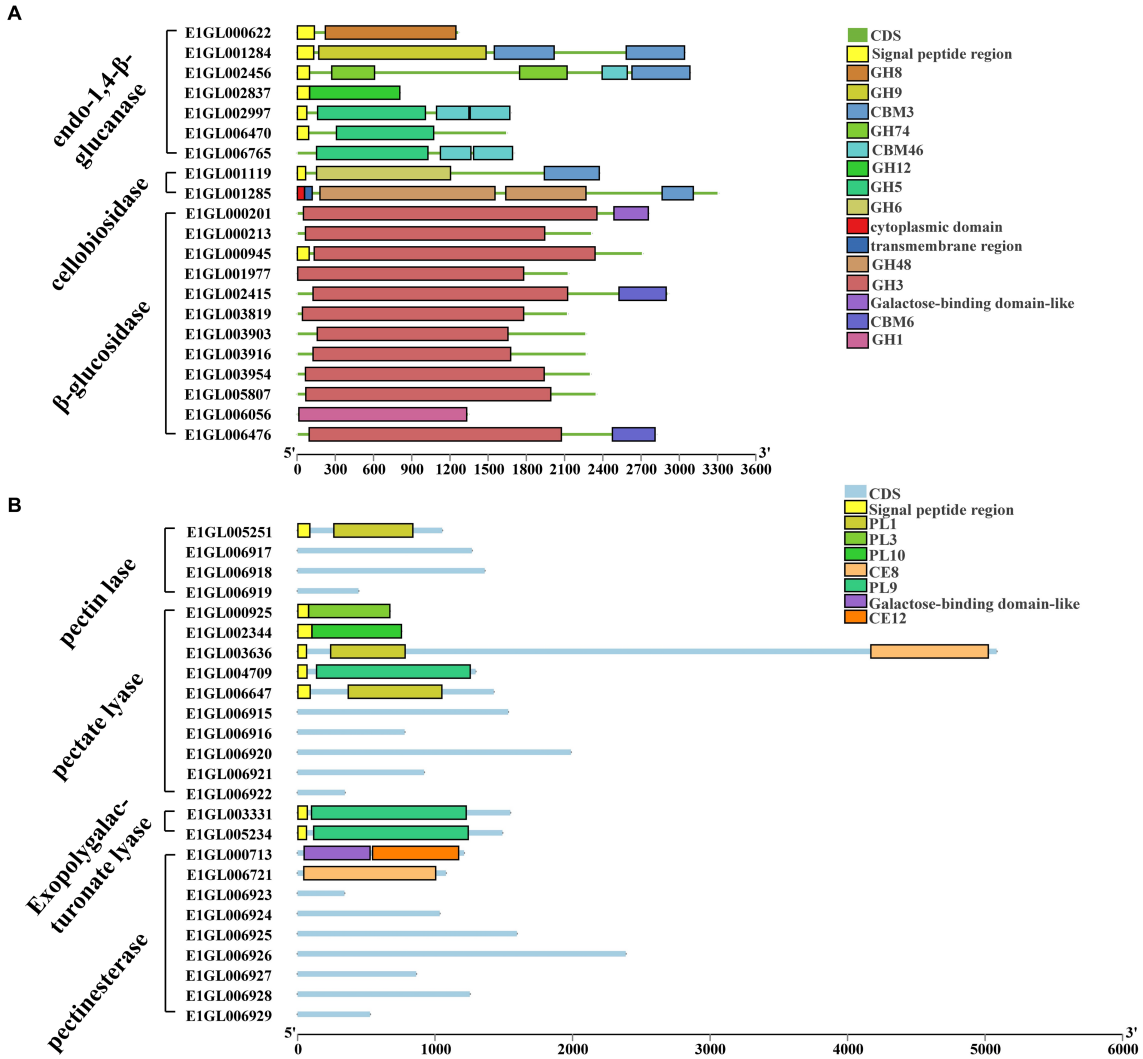
Cellulose and pectin degrading enzyme gene ID and CAZyme family/domain. **(A)** Cellulose degrading enzyme. Seven genes were annotated as endo-1,4-beta-D-glucanase. Two genes were annotated as cellobiosidase. There are 12 genes annotated as beta-glucosidase. **(B)** Pectin degrading enzymes. Four genes were annotated to pectin lyases. Ten genes were annotated to pectate lyases. Two genes were annotated as exo-polygalacturonate lyases. Nine genes were annotated as pectate esterases.

Complete cellulose degradation necessitates the presence of endo-1,4-beta-D-glucanase, cellobiohydrolase, and beta-glucosidase enzymes (Figure 2A). Endo-1,4-beta-D-glucanase enzymes randomly cleave O-glycoside bonds. Among the annotated genes, seven were identified as endo-1,4-beta-D-glucanases, with six of them containing signal peptides and one being an intracellular protein. These seven proteins belong to five distinct GH families: GH5, GH8, GH9, GH12, and GH74. Notably, E1GL002997 and E1GL006765 of the GH5 family possess a CBM46 domain at their C-terminal ends. Cellobiosidase (exo-cellulase) catalyzes the processive hydrolysis of cellulose chains toward the crystalline region, generating cellobiose. Two genes, E1GL01119 and E1GL01285, were annotated as cellobiosidases. E1GL01119 belongs to GH6 and is linked to a CBM3 domain at its C-terminal, while E1GL001284, E1GL002456, E1GL001119, and E1GL001285 are all associated with a CBM3 domain at their C-terminal ends. Beta-glucosidase (BG) acts on cellobiose, producing glucose as the final product. Among the annotated genes, 12 were identified as beta-glucosidases, with only E1006056 belonging to the GH1 family and the rest belonging to the GH3 family. Notably, E1GL00945 possesses a signal peptide for extracellular secretion, whereas E1GL002415 and E1GL006476 possess a CBM6 domain at their C-terminal ends. Additionally, E1001978 and E1006424 were annotated as cellobiose phosphorylases (CBP), which differ from beta-glucosidases as they act on cellobiose through the phosphorolysis pathway, resulting in glucose and glucose-1-phosphate as the final products. CBP belongs to the GH94 family and exhibits strict substrate specificity.

#### 3.1.2. Hemicellulose degradation

**Figure 3:**
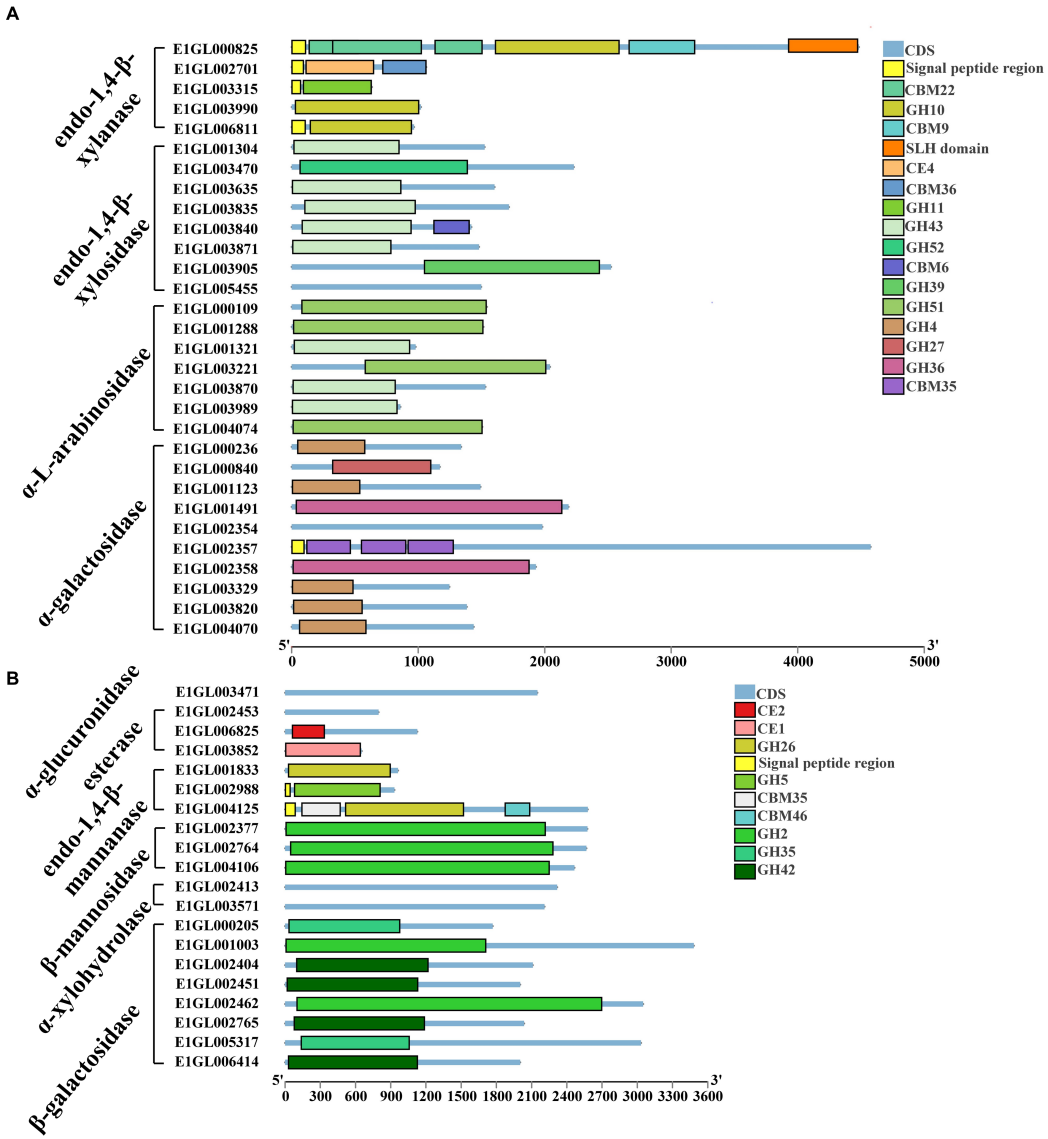
Hemicellulose degrading enzyme gene ID and CAZyme family/domain. **(A)** Five genes were annotated as beta-1,4-endo-D-xylanase. Eight genes were annotated as exo-1,4-beta-xylosidase. Seven genes were annotated as alpha-L-arabinosidase. Ten genes were annotated as alpha-galactosidase. **(B)** One gene was annotated as alpha-glucuronidase. Three genes were annotated as acetyl xylan esterases. Three genes were annotated as endo-1,4-beta-mannanase. Three genes were annotated as beta-mannosidase. Two genes were annotated as alpha-xylosidases. Two genes were annotated as alpha-xylohydrolase. Eight genes were annotated as beta-galactosidases.

*Paenibacillus pabuli* E1 is equipped with the necessary enzymes for complete xylan hydrolysis (Figure 3). Five genes were annotated as beta-1,4-endo-D-xylanases, with four of them possessing signal peptides. E1GL000825, E1GL003990, and E1GL006811 belong to the GH10 family, while E1GL003315 belongs to the GH11 family. E1GL000825 is linked to CBM9 and CBM22 domains at its C-terminal end, and E1GL002701 is connected to the CBM36 domain. Moreover, eight genes were annotated as exo-1,4-beta-xylosidases. E1GL003470 and E1GL003905 belong to the GH52 and GH39 families, respectively, while the remaining six genes belong to the GH43 family. Natural xylan comprises various substituent groups on the main chain and side chain sugar groups, including arabinoyl and glucuronyl. Seven genes were annotated as alpha-L-arabinosidases, all of which belong to the exo-glycosidases. Specifically, E1GL000109, E1GL001288, E1GL003221, and E1GL004074 are classified under the GH51 family, while E1GL001321, E1GL003870, and E1GL003989 belong to the GH43 family. Alpha-galactosidase catalyzes the hydrolysis of galactose residues from the side chains of xylans or galactomannans at non-reducing ends. This study identified 10 annotated genes for alpha-galactosidase, with E1GL002357 and E1GL003820 possessing signal peptides. These enzymes belong to the GH4, GH27, and GH36 families. E1GL003471 was the sole annotated alpha-glucuronidase in *P. pabuli* E1, belonging to the GH67 family. Additionally, hemicellulose is typically acetylated, and the presence of acetyl groups limits the action of xylan-degrading enzymes. E1GL002453 and E1GL006825 were annotated as acetyl xylan esterases, with E1GL006825 belonging to the CE2 domain. Furthermore, E1GL003852 was annotated as a ferulic acid esterase responsible for cleaving the ester bond between ferulic acid and arabinose residues.

The backbone of mannan consists of beta-1,4-linked mannose or a combination of mannose and glucose residues, often substituted by alpha-1,6-linked galactose. The main enzymes involved in mannan degradation are beta-mannanase and beta-mannosidase, along with additional enzymes such as alpha-galactosidase and acetyl mannan esterase to remove side groups from the mannan main chain. Three genes, E1GL001833, E1GL002988, and E1GL004125, were annotated as endo-1,4-beta-mannanases. E1GL001833 and E1GL004123 belong to the GH26 family, while E1GL002988 belongs to the GH5 family. These three genes are linked to different CBM domains at their C-terminal ends. Additionally, E1GL002377, E1GL002764, and E1GL004106 were annotated as beta-mannosidases, all belonging to the GH2 family.

Xyloglucan serves as the primary hemicellulose in the primary cell walls of dicots and non-graminous monocots. Two genes, E1GL002413 and E1GL003571, were annotated as alpha-xylosidases, and classified under the GH31 family. GH31 enzymes catalyze the hydrolysis of terminal unsubstituted xylosides at the reducing ends of xylogluco-oligosaccharides. Eight genes were annotated as beta-galactosidases, distributed across the GH2, GH35, and GH42 families. Specifically, E1GL001003, E1GL002404, and E1GL002462 belong to the GH2 family. Moreover, E1GL001003 and E1GL002404 are linked to the CBM6 domain at their C-terminal ends, while E1GL002462 is linked to the CBM51 domain.

#### 3.1.3. Pectin degradation

The *P. pabuli* E1 genome contains 10 pectate lyases (PGL) and four pectin lyases (PMGL) belonging to the polysaccharide lyases (PL) family, distributed across PL1, PL3, PL9, and PL10 (Figure 2B). Among these, only the pectin lyase E1GL005251 belongs to the PL1 family, while the others lack PL1 domain characteristics. The pectate lyases E1GL000925, E1GL002344, E1GL003636, E1GL004709, and E1GL006647 all possess signal peptides. E1GL000925, E1GL002344, and E1GL004709 belong to PL3, PL10, and PL9, respectively, while E1GL003636 and E1GL006647 belong to PL1. Additionally, E1GL003331 and E1GL005234 were annotated as exo-polygalacturonate lyases, both with signal peptides and classified under PL9. Nine genes were annotated as pectate esterases, with E1GL000713 and E1GL006647 belonging to CE12 and CE8, respectively, while the remaining genes lacked a clear domain match.

#### 3.1.4. Lignin degradation

The potential lignin-degrading enzymes identified in *P. pabuli* E1 are MnP (from E1GL004832) and laccase (from E1GL004895). The E1GL004832 showed 100% similarity to the reported manganese catalase (WP_076288188.1) from *Lactobacillus plantarum* when analyzed using the blastp tool. Manganese catalase exhibits a hexameric structure that is stabilized through extensive contacts between subunits. Each subunit contains a dimanganese active site. Similarly, the sequence E1GL004895 exhibited 100% similarity to the reported polyphenol oxidase (CAH1208948.1) from *Paenibacillus* sp. JJ-223 when analyzed using blastp. The COG database annotation identifies it as a Copper oxidase (laccase).

### 3.2. Growth in different polysaccharide mediums

*Paenibacillus pabuli* E1 exhibited growth in polysaccharide media supplemented with carboxymethyl cellulose (CMC), xylan, and pectin. However, the growth was limited when cellulose powder was used, and no growth was observed when filter paper was used as the sole carbon source (Table 1). Lignin, a non-polysaccharide compound, was also tested, and growth was observed in a minimal medium supplemented with lignin. To investigate the potential induction of cellulase, filter paper or CMC was added to the LB activation culture medium. The results showed that no reducing sugar activity was detected regardless of whether filter paper or CMC was pre-added to the activation medium. The disintegration of filter paper was observed to varying degrees after incubating with the supernatant and cell insoluble fraction for 4 h, with the control group being PBS solution (Supplementary Figure S1). The cell insoluble fraction showed a stronger disintegration effect on filter paper, but no reducing sugar was detected in either case.

**Table 1:** Growth in polysaccharide medium.

Polysaccharide type	Growth
No sugar	−
Cellulose powder	+
CMC	+
Filter paper	−
Xylan	+
Pectin	+

### 3.3. Enzyme assay

The endoglucanase (CMCase) activity of *P. pabuli* E1 was measured to be 5.37 U/mL, while the beta-glucosidase activity was 4.60 U/mL. No reducing sugars were detected in the filter paper experiments. In the degradation of xylan, the concerted action of complex enzymes is required, with beta-xylanase and beta-xylosidase being the most crucial enzymes involved. These enzymes are capable of degrading the main chain structure of xylan. Therefore, in the present experiment, the activities of beta-xylanase and beta-xylosidase were tested. The beta-xylanase and beta-xylosidase activities were determined to be 11.05 U/mL and 4.16 U/mL, respectively. Pectate lyase and pectin lyase activities reached 8.19 U/mL and 2.43 U/mL, respectively. Lignin, a highly complex aromatic polymer, poses challenges for degradation due to its intricate structure. The activities of laccase and MnP in *P. pabuli* E1 were measured to be 1.87 U/mL and 4.30 U/mL, respectively. Laccase and MnP are responsible for lignin degradation in bacteria. All enzyme activity values are shown in Table 2.

**Table 2:** Enzymatic activities detected.

Enzyme type	Enzyme activity (U/mL)
Endoglucanase	5.37
Beta-glucosidase	4.60
FPAase	-
Beta-xylanase	11.05
Beta-xylosidase	4.16
Pectate lyase	8.19
Pectin lyase	2.43
Laccase	1.87
MnP	4.30

### 3.4. Solid-state fermentation

**Figure 4:**
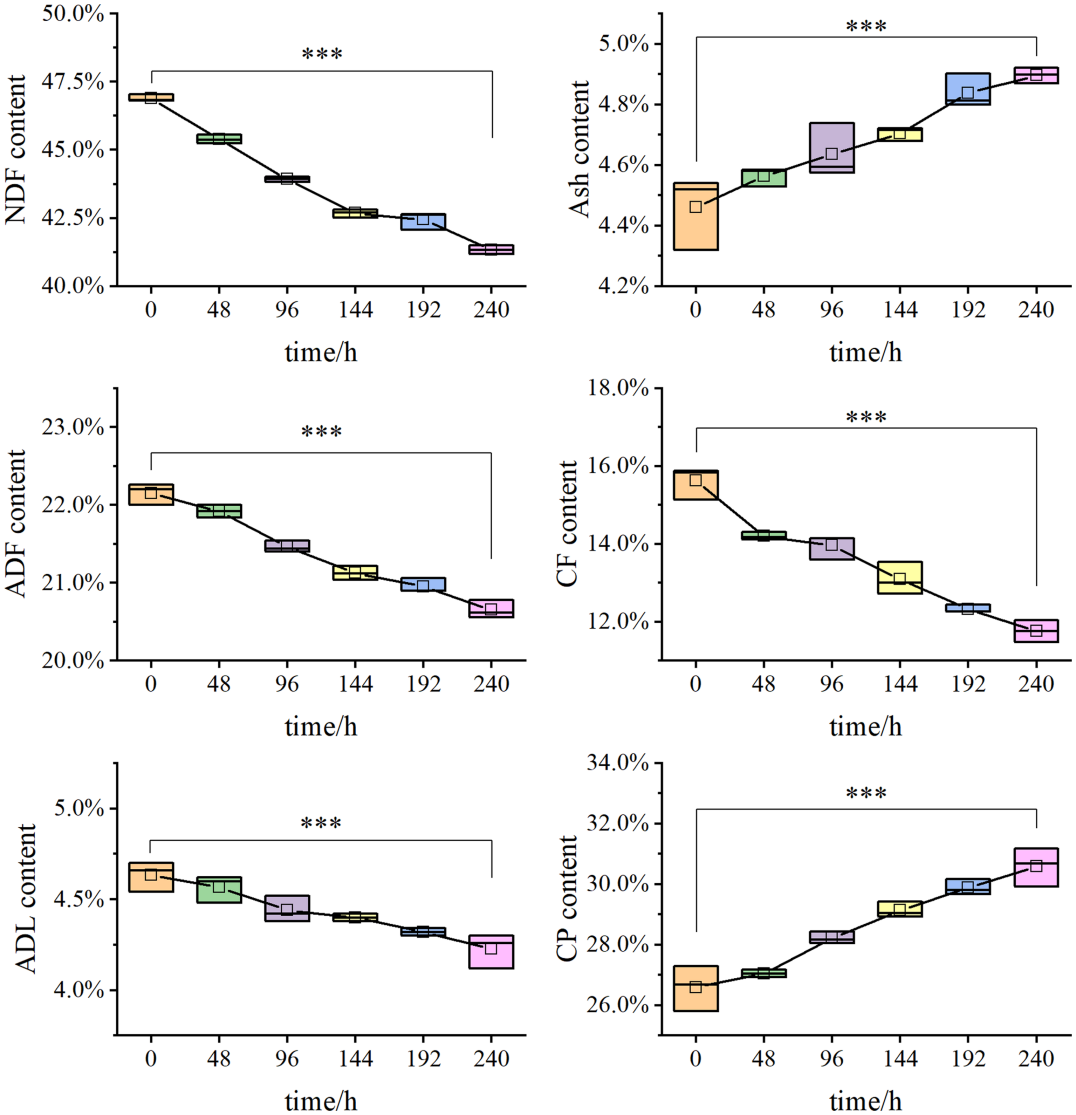
Degradation rates of cellulose, hemicellulose, and lignin in solid-state fermentation. The NDF content decreased from 46.9% to 41.33%, the ADF decreased from 22.15% to 20.65%, and ADL decreased from 4.63% to 4.23%. Ash increased from 4.46% to 4.90%. CF reduced from 15.62% to 11.76%. CP increased from 26.59% to 30.59%. *** Indicates *p*-value < 0.001, which is considered extremely significant.

**Figure 5:**
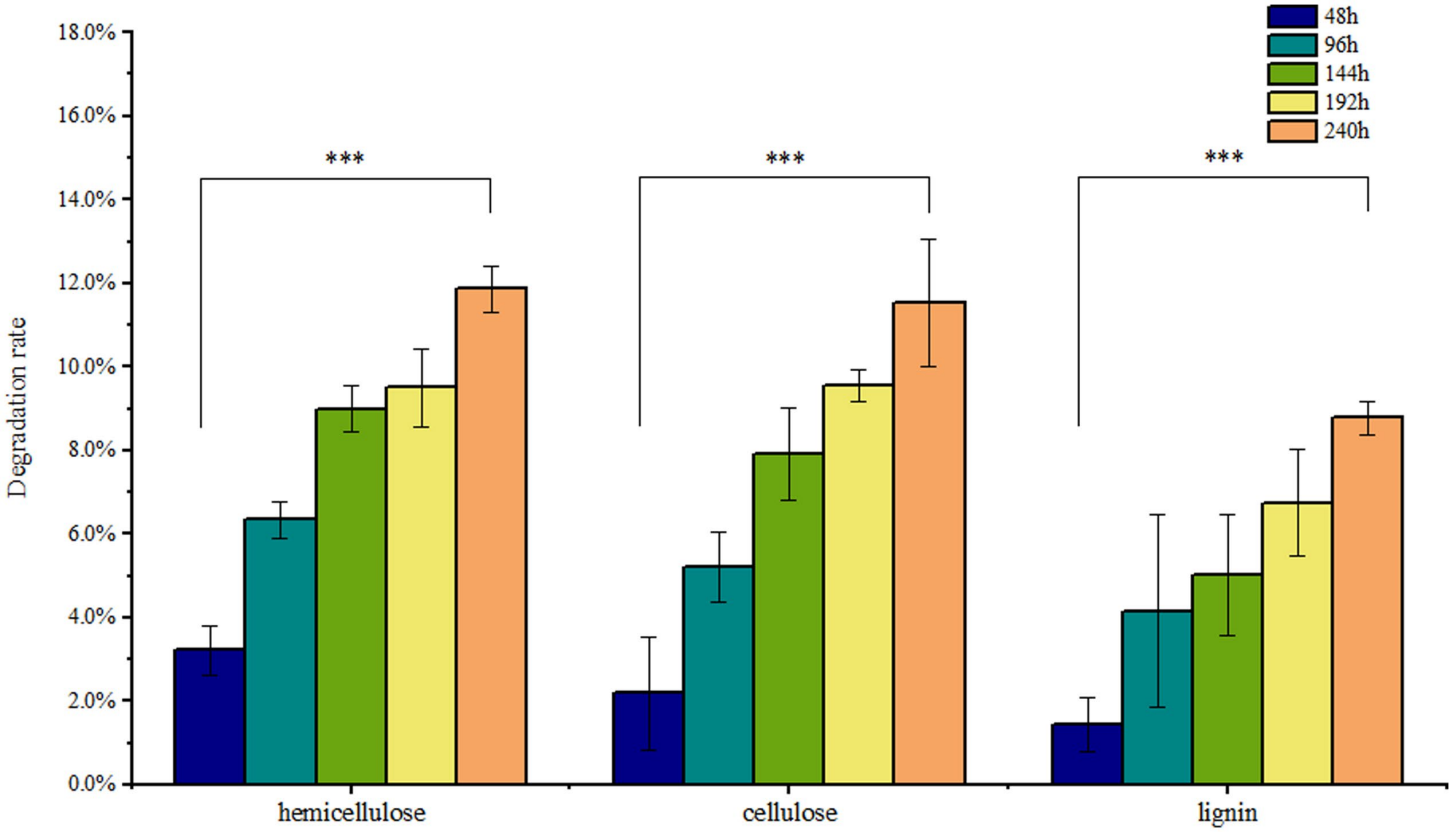
Nutrients in DDGS at different fermentation times. After 240 h of DDGS fermentation, the degradation rates of hemicellulose, cellulose, and lignin were 11.86%, 11.53%, and 8.78%, respectively (*p* < 0.001). *** Indicates *p*-value < 0.001, which is considered extremely significant.

Solid-state fermentation was employed to reduce NSPs such as lignocellulose. After 7 days of fermentation, the neutral detergent fiber (NDF) content in DDGS decreased from 46.9% to 41.33%, while the acid detergent fiber (ADF) decreased from 22.15% to 20.65%. Similarly, the acid detergent lignin (ADL) content decreased from 4.63% to 4.23%. These results demonstrate that *P. pabuli* E1 effectively reduced the lignocellulosic content of DDGS through fermentation. The ash content in DDGS increased from 4.46% to 4.90%, potentially due to the addition of inorganic salt ions during the preparation of *P. pabuli* E1. *CF*, which serves as an important parameter in feed formulation, decreased from 15.62% to 11.76%. Furthermore, CP content in DDGS increased from 26.59% to 30.59% (Figure 4). NDF comprises cellulose, hemicellulose, lignin, and ash, while ADF contains the latter three components. By calculating the differences between NDF, ADF, ADL, and ash, the content of cellulose, hemicellulose, and lignin can be obtained. After 240 h of DDGS fermentation (*p* < 0.001), the degradation rates of hemicellulose, cellulose, and lignin were determined to be 11.86%, 11.53%, and 8.78%, respectively (Figure 5).

## 4. Discussion

### 4.1. Cellulose degradation

The disintegration of filter paper without the presence of reducing sugars suggests that *P. pabuli* E1 may produce cellulose accessory proteins that disrupt the crystalline structure of cellulose without exhibiting hydrolase activity. Previous studies have reported the production of auxiliary proteins such as expansin and swollenin by some microorganisms. These proteins loosen the cellulose structure by breaking hydrogen bonds between microfibrils, thus increasing the accessibility of cellulase to the substrate (Hendriks and Zeeman, 2009). Kim et al. (2009) demonstrated that expansin from *Bacillus subtilis* synergistically increased cellulase activity by 5.7-fold. Therefore, it is speculated that the disintegration of filter paper observed in *P. pabuli* E1 may be caused by an auxiliary protein that disrupts the network structure of the cellulose surface without detectable reducing sugars but significantly enhances cellulase hydrolytic activity. Expansins are present in various plants and microorganisms, and they share homology with the GH45 family of glycoside hydrolases. However, expansins lack a complete catalytic mechanism, and no hydrolytic activity has been detected (Georgelis et al., 2015). Expansins from *B. subtilis* do not possess hydrolytic activity but contribute to the loosening of cellulose structure, thereby facilitating cellulose biotransformation (Kim et al., 2009). Swollenin, a non-enzymatic protein with high sequence similarity to expansins, has also been found in *Trichoderma reesei*. Swollenin swells cotton fibers without producing detectable reducing sugars (Chen et al., 2010). The application of swollenin effectively alleviates the hindering effect of the crystallization region of the substrate on the hydrolysis reaction and increases the affinity between the enzyme and the substrate, thus enhancing cellulase hydrolysis activity (Saloheimo et al., 2002). Unfortunately, the genome of *P. pabuli* E1 did not match any proteins similar to the reported auxiliary proteins. There are few studies on auxiliary proteins in bacteria, and it is not ruled out that there are novel auxiliary proteins.

Cellulolytic enzymes are essential to break down cellulose thoroughly, although the role of accessory proteins is indispensable. Fungi, known for their potent ability to degrade lignocellulose, have been extensively studied as the primary group of microorganisms involved in plant biomass degradation. This is attributed to their remarkable enzyme secretion capacity and the robustness of their degradation enzymes. Among the cellulose-degrading microorganisms, *A. Niger* and *T. reesei* are the most extensively investigated. The study revealed the complete secretomes of *A. Niger* and *T. reesei* are involved in lignocellulose degradation. These secretomes include the well-known GH7 and GH6 cellobiohydrolases, GH5 endoglucanases, beta-glucosidases, as well as additional enzymes that target other components of the plant cell wall (Borin et al., 2015). A total of 430 CAZymes were annotated in *T. harzianum*. These included 259 GHs, 101 GTs, 6 PLs, 22 CEs, 42 AAs and 46 CBMs (Ferreira Filho et al., 2017).

When compared to fungi, bacteria generally exhibit lower cellulase activity. However, the characterization of novel enzymes has increasingly focused on bacterial sources due to the high specific activity (Obeng et al., 2017). Among the bacterial strains isolated for their ability to degrade crystalline cellulose, the majority belong to specific lineages within the four major phyla: Actinobacteria, Firmicutes, Proteobacteria, and Bacteroidetes (Koeck et al., 2014). The primary cellulase-producing bacteria are often from the genus *Bacillus*, such as *B. licheniformis* and *B. subtilis*, as well as from the genus *Clostridium*. Anaerobic bacteria play a significant role among cellulose-degrading bacteria. In anaerobic bacteria, cellulases are typically associated with the bacterial surface in the form of cellulosomes. Certain anaerobic members of the *Clostridia* group are known to produce cellulosomes, which are highly efficient multienzyme complexes attached to the outer surface of bacterial cell (Wilson, 2008). This arrangement allows for enzyme recycling and rapid assimilation of hydrolytic products. Whole genome sequencing has led to the characterization of key components of cellulosomes. The structure of cellulosomes can vary in complexity among different bacterial species. The most prominent enzymes integrated into cellulosomes typically belong to GH48, GH9, and GH5 families, which are involved in cellulose degradation (Artzi et al., 2017). However, it should be noted that the genome of *P. pabuli* E1 does not contain the structural skeleton proteins typically associated with cellulosomes.

In contrast, aerobic bacteria are capable of secreting extracellular enzymes involved in cellulose degradation. Members of the *Bacillus* and *Paenibacillus* exhibit significant cellulolytic capability, which is attributed to the presence of a wide range of glycoside hydrolase (GH) enzymes in their genomes (Ma et al., 2020). For instance, *B. velezensis* LC1 demonstrated promising cellulase activity. Carbohydrate-active enzyme annotation revealed 136 genes associated with CAZy families. The cellulase activities of strain LC1 were then determined. The endoglucanase activity was measured as 0.689 ± 0.011 U/mL on day 1 and increased to 0.752 ± 0.013 U/mL on day 6. The exoglucanase activity ranged from 0.359 ± 0.016 U/mL to 0.385 ± 0.022 U/mL (Li et al., 2020).

The agricultural waste hydrolyzing capabilities of *Paenibacillus dendritiformis* CRN18 were investigated. The strain exhibited enzyme activities for exo-glucanase, beta-glucosidase, beta-glucuronidase, endo-1,4-beta-xylanases, arabinosidase, and alpha-galactosidase at levels of 0.1, 0.3, 0.09, 0.1, 0.05, and 0.41 U/mL, respectively (Srivastava et al., 2020). Genomic analysis of *Paenibacillus lautus* BHU3 strain predicted 6,234 protein-coding genes, with 316 genes associated with sugar metabolism. This analysis suggests an important role in enhancing cellulolytic properties (Kim et al., 2018). Similarly, *Paenibacillus* sp. strains IHB B 3415, a cellulase-producing psychrotrophic bacterium, contained 1,011 genes assigned for carbohydrate metabolism, including 16 genes predicted for cellulases, supporting its cellulose degradation capabilities (Dhar et al., 2015). Among the examined isolates, *Paenibacillus* sp. O199 demonstrated the highest efficiency for cellulose deconstruction. Its genome contained 476 genes associated with CAZyme families, including 100 genes coding for GHs potentially involved in cellulose and hemicellulose degradation (Lopez-Mondejar et al., 2016). *Paenibacillus pabuli* E1 possesses multiple types of endo-1,4-beta-D-glucanases with different substrate affinities. The GH8 family exhibits broad substrate specificity and catalyzes various polysaccharides such as carboxymethylcellulose (CMC), chitosan, barley-beta-glucan, lichenin, and xylan sugars (Ontanon et al., 2019). GH74 is considered an important family of endoglucanases, but recent studies have shown high specificity for xyloglucan (Wang et al., 2022). CBM46 enhances the enzyme’s affinity for cellulose, and some studies have indicated that the catalytic activity of enzymes depends entirely on the CBM46 domain (Liberato et al., 2016).

Cellobiosidases are classified into CBH I and CBH II based on the reducing properties of the bound chain ends. GH6 is a typical representative of CBH II enzymes that act on the non-reducing end of cellulose molecules. Most GH6 enzymes exhibit processive catalysis, and their catalytic domains have a short connecting peptide to the CBM domain, which is essential for enzyme activity (Irwin et al., 1998). E1GL01285, belonging to the GH48 domain, is a CBH I enzyme that acts on the reducing end of cellulose and is present in many bacterial cellulase systems. GH48 enzymes have inherently low cellulolytic activity but exhibit strong synergy with GH9 endoglucanases, even at low ratios. CBM3 can enhance cellulose adsorption capacity, which is beneficial for the catalysis of endo-cellulases and cellobiosidases.

The presence of endo-1,4-beta-D-glucanases and cellobiosidases is crucial for the initial degradation of cellulose. Endo-glucanases randomly bind to non-crystalline regions of microfibrils, generating new reducing ends, while exo-cellulases bind to the reducing or non-reducing end of a single cellulose chain and processively degrade the crystalline region. Studies have indicated that the loss of exo-cellulase activity is a major factor in the reduced rate of enzymatic cellulose hydrolysis (Wang et al., 2006). Although cellulose is the primary resource in feed for ruminant livestock, monogastric animals such as pigs and chickens have limited ability to degrade cellulose. *Paenibacillus pabuli* E1 possesses the necessary enzymes to break down cell walls, thereby releasing nutrients and significantly improving feed nutrient absorption rates.

### 4.2. Hemicellulose degradation

Xylan, as an anti-nutritional factor present in feed, poses obstacles to the absorption and utilization of nutrients in livestock (Dikeman and Fahey, 2006). Among the constituents of feed ingredients, hemicellulose primarily comprises xylan, mannan, and xyloglucan (de Vries and Visser, 2001). When the culture temperature was raised to 37°C and the initial pH was set at 8.5, the xylanase activity of *Paenibacillus glycanilyticus* X1 peaked at 189.6 mU after 72 h (Wang and Liang, 2021). Another study focused on *Paenibacillus* sp. BL11 strain, which exhibited the highest enzyme activity at an initial pH of 8 and a culture temperature of 37°C. This strain displayed greater xylanase activity under neutral to slightly alkaline conditions and at moderate to high temperatures (Ko et al., 2010). The endo-beta-1,4-xylanase derived from *Paenibacillus curdlanolyticus* B-6, belonging to the GH10 family, was found to possess CBM22 and CBM3 domains at its C-terminus (Sermsathanaswadi et al., 2017). Additionally, a novel GH6 cellobiohydrolase from the same strain, *Paenibacillus curdlanolyticus* B-6, demonstrated high substrate specificity toward amorphous cellulose and lower specificity toward crystalline cellulose. However, this enzyme did not exhibit activity on substitution substrates such as carboxymethyl cellulose and xylan (Baramee et al., 2017). *Paenibacillus* sp. LS1 exhibited the ability to utilize different types of xylans. Genome analysis revealed a comprehensive set of xylan-active CAZymes, indicating its capacity for efficient degradation of xylan (Mukherjee et al., 2023). Moreover, *Paenibacillus* sp. strains DA-C8 completely degraded 1% beechwood xylan within 4 days under anaerobic conditions, while it was capable of growth on xylan medium under aerobic conditions (Chhe et al., 2021a,b). In the case of *Paenibacillus physcomitrellae* XB, different xylan degradation abilities were observed for various substrates such as corncob xylan, oat spelled xylan, and wheat flour arabinoxylan. The bifunctional enzymes Ppxyl43A and Ppxyl43B identified in this strain hold promise for xylan biomass conversion (Zhang et al., 2021).

*Paenibacillus pabuli* E1 exhibits higher levels of beta-xylanase and beta-xylosidase compared to similar strains (Ghio et al., 2020). Xylanases are commonly classified into two families. GH10 xylanases have a high molecular weight and possess four or five substrate binding sites, while GH11 xylanases catalyze xylans with a minimum of three adjacent xyloses without substituents (Mendis et al., 2016; Chang et al., 2017). GH11 xylanases demonstrate higher specificity, with superior hydrolytic activity toward long-chain xylan compared to GH10 xylanases. The presence of CBM9 and CBM22 domains enhances the adsorption capacity of xylan. CBM22 aids in delivering xylan to the adjacent catalytic domain of GH10 by promoting binding to xylan, whereas CBM9 enhances xylan degradation by binding to cellulose (Tanimoto et al., 2016). CBM36 is a novel carbohydrate-binding module that exhibits Ca^2+^-dependent affinity for xylan. Additionally, the CBM2 domain exhibits broad substrate recognition and can bind xylan, cellulose, and chitin (Tanimoto et al., 2016). Xylanases are responsible for cleaving the glycosidic linkages in the xylan backbone, leading to the generation of xylooligosaccharides. Beta-1,4-xylosidase further hydrolyzes xylooligosaccharides into xylose, which is crucial for complete xylan degradation. The activity of beta-1,4-xylosidase derived from Bacillus sp. in the GH52 family gradually decreases as the length of the main chain increases (Teramoto et al., 2021). Alpha-L-arabinosidase acts on alpha-nitrophenol-furan arabinoside or branched arabinan. Some xylanases cannot degrade glycosidic bonds between xylose units with substituted side chains, while others can only hydrolyze side chain substituents on xylooligosaccharides. Alpha-L-arabinosidase is classified into the GH43 and GH51 families. The presence of metal ions enhances the enzyme activity of the GH43 family, whereas the GH51 enzyme hydrolyzes alpha-1,2 or alpha-1,3-linked arabinofuranose residues. The synergy between GH51 and GH43 enzymes improves the debranching efficiency of arabinoxylans (Koutaniemi and Tenkanen, 2016). Alpha-glucuronidase, as the rate-limiting enzyme in xylan degradation, plays a crucial role in the biodegradation of xylan hemicellulose. It acts specifically on small molecules of 4-O-methylglucurono-oligosaccharides with residues, hydrolyzing the side chain of the non-reducing end of 4-O-methylglucuronic acid (de Wet et al., 2006). *Paenibacillus* sp. TH501b isolates from soil samples contain alpha-glucuronidase. This enzyme is most active at pH 6.0–7.0 and 30°C (Iihashi et al., 2009). Acetyl xylan esterase primarily acts on the 2- or 3-position O-acetyl groups of xylose residues in acetylated xylan. The CE2 domain exhibits strong specificity for the 4-position acetyl group of xylopyranose residues. The degree of 2-O- and 3-O-acetyl substituents in xylan is challenging to determine, and the release of O-acetyl groups reduces pH, which inhibits fermenting microorganisms (Basen et al., 2014). Ferulic acid, found as monomers and dimers linked to arabinoxylan residues, is enzymatically hydrolyzed from the hemicellulose substrate, loosening the cell wall structure and increasing the rate of cellulose degradation (Krueger et al., 2008).

Mannan, another type of anti-nutritional factor present in plant-based feedstuffs such as soybean meal, rapeseed meal, sesame meal, and corn, can be degraded by beta-mannanase. Among them, the highest content of beta-mannan in soybean meal is 1.1%–1.3% (Seo et al., 2015). Beta-mannanase facilitates the release of nutrients encapsulated in the cell wall and reduces chyme viscosity, thereby improving nutrient absorption in animals. Enzyme supplementation enhances feed digestion, utilization, and animal production performance (Chauhan et al., 2012). CBM6-associated beta-galactosidases not only adsorb amorphous cellulose but also bind to beta-1,3-glucan, beta-1,3/1,4-glucan, and beta-1,4-glucan. Although research on CBM51 is limited, its members demonstrate specificity for eukaryotic glycans (Gregg et al., 2008).

Xyloglucan is initially hydrolyzed by endo-1,4-beta-glucanase, producing xyloglucan oligosaccharides (XGOs) that are further hydrolyzed by beta-galactosidase, releasing galactose monomers. Alpha-xylosidase removes xylosyl residues in oligosaccharides, while beta-glucosidase hydrolyzes glucose residues in the xyloglucan backbone (Attia and Brumer, 2016). The structural complexity of hemicelluloses, with their varied main chains and modified side chain groups, poses challenges for complete degradation. *Paenibacillus pabuli* E1 possesses a repertoire of enzymes targeting both the main chains and side chains, which are essential for efficient hemicellulose degradation. The *P. pabuli* E1 genome contains enzymes that degrade different types of xylan, which is also helpful for eliminating xylan in other cereals not limited to those in DDGS.

### 4.3. Pectin degradation

Pectin is composed of D-galacturonic acid units connected via alpha-1,4-glycosidic linkages, with side chains comprising rhamnose, arabinose, galactose, and xylose. Pectin is classified into four types: pectic acid, pectinic acid, pectin, and protopectin. In *Paenibacillus amylolyticus* 27C64, a comprehensive analysis identified a total of 314 putative carbohydrate-active enzymes (CAZymes) distributed among 108 distinct families. Further investigation of the culture supernatants revealed the presence of pectinase activities (Keggi and Doran-Peterson, 2019). An alkaline pectate lyase gene derived from *Paenibacillus polymyxa* KF-1 was also studied. This gene encodes a protein consisting of 449 amino acid residues and belongs to the polysaccharide lyase family 9 (PL9). The optimal conditions for its enzymatic activity were determined to be at pH 10.0 and a temperature of 40°C (Zhao et al., 2018; Yuan et al., 2019).

Structurally, lyases from PL1, PL3, and PL9 families exhibit a parallel beta-helix conformation, while the PL10 family adopts an (alpha/alpha)3-barrel structure. Galacturonic acid plays a vital role in maintaining the pectin structure, making the degradation of polygalactose essential. Additionally, nine pectin esterases promote the hydrolysis of pectin esters by removing methyl groups, thus facilitating the production of pectinic acid. PMGL, on the other hand, directly degrades high-methoxyl pectin without the need for esterases to remove methyl ester groups. Most pectin methylesterases (PGLs) degrade polygalacturonic acid and low-methoxyl pectin but exhibit less activity toward high-methoxyl pectin. In animals, the absence of endogenous pectinases hampers the digestion of pectin, making pectinase supplementation crucial for improving crude fiber utilization (Sharma et al., 2012; Lima et al., 2017; Yang et al., 2020). Pectate lyase and pectin lyase act on the alpha-1,4-glycosidic bonds of pectin or pectinic acid, generating unsaturated pectin oligosaccharides without producing highly toxic methanol. Pectinase finds applications in various industries such as food fermentation, paper biopulping, animal feed, and environmental protection (Kohli and Gupta, 2015).

### 4.4. Lignin degradation

Microbial degradation of lignin represents a promising approach for mitigating its deleterious effects. Among microorganisms, fungi possess a diverse array of lignin depolymerase systems, with significant research attention directed toward white-rot and brown-rot fungi (Wang et al., 2019). However, our understanding of bacterial ligninases remains limited, despite their potential importance in lignin degradation. *Paenibacillus* sp. DLE-14, isolated from plant roots, demonstrates the potential to degrade lignin. Lignin degradation is commonly associated with the action of two prominent enzymes, namely laccase and manganese peroxidase (MnP). However, the presence and activity of these enzymes in the *Paenibacillus* species have received limited research attention. To date, only one study has reported the purification of MnP from *Paenibacillus* sp., revealing an enzyme activity of 4.3 U/L under optimal conditions (De et al., 2009).

MnP activity is primarily dependent on Mn^2+^ ions, which are oxidized to Mn^3+^ and form complexes with glycolic acid and oxalic acid present in the system. MnP exhibits a low oxidation/reduction potential and selectively degrades phenolic lignin (Xu et al., 2018). Laccase, belonging to the blue multi-copper oxidase family, is a polyphenol oxidase with a broad range of substrates, including phenols, aromatic amines, carboxylic acids, and steroids. It catalyzes the oxidation of phenols by generating phenoxy radicals, thereby promoting lignin degradation (McMahon et al., 2007).

Laccases are found in fungi, plants, and other bacteria. However, most laccases cannot directly oxidize non-phenolic compounds due to their high redox potential compared to the “normal hydrogen electrode” (NHE), whereas the redox potential of laccases is lower than 0.8 V (Canas and Camarero, 2010). Nevertheless, there are two basidiomycetes known to produce laccases with high redox potential (Hernández-Martínez et al., 2017, 2018). White-rot fungi are generally more efficient in lignin degradation, but their enzymes are susceptible to loss of activity under extreme temperature and pH conditions. However, heat-resistant laccases produced by *Pycnoporus sanguineus* CS43 (LacI and LacII) have demonstrated high resistance to organic solvents such as acetonitrile, ethanol, and acetone (Ramírez-Cavazos et al., 2014). Enzymes with superior tolerance are more suitable for industrial and agricultural applications. In comparison to fungal laccases, bacterial laccases exhibit high activity at elevated temperatures, alkaline pH, and high chloride and copper ion concentrations, making them compatible with various industrial processes (Ausec et al., 2011; Chandra and Chowdhary, 2015). Bacteria may also possess the ability to modify lignin and release smaller aromatic compounds that can be imported into cells and metabolized through aromatic catabolism (Brown and Chang, 2014). Bacterial enzymes show significant potential in lignin degradation and have become candidates for commercial production. While *P. pabuli* E1 enzymes have lower oxidation/reduction potentials and inferior lignin degradation ability compared to fungi, bacteria’s strong environmental adaptability and biodiversity have sparked interest in bacterial lignin degradation research (Wang X. et al., 2018).

### 4.5. Solid-state fermentation

CF refers to the content of fiber in a sample, and it is important to note that the use of acid–base reagents during sample preparation may lead to the destruction of some cellulose and hemicellulose, resulting in a measured value smaller than the actual value. ADF comprises cellulose, lignin, and a small amount of acid-insoluble silicate ash, and there is a strong correlation between ADF and CF in DDGS (Pekel et al., 2013; Liu et al., 2018). DDGS typically contain varying amounts of NDF, ADF, CF, and ADL (Kannadhason et al., 2009). The NDF content is generally higher than the ADF content, indicating a higher hemicellulose content in DDGS (Sharma et al., 2016). Solid-state fermentation has been shown to effectively reduce the content of NSPs in DDGS and improve its nutritional value. Studies have demonstrated that solid-state fermentation using *B. subtilis* and *L. plantarum* can reduce lignin, ADF, and NDF contents by 1.1%, 5%, and 20.4% respectively (Wang C. et al., 2018). Supplementation of cornmeal with *Lactobacillus plantarum* can reduce *CF* content by approximately 77% (Terefe et al., 2021).

Fungi are commonly employed in the fermentation of DDGS. Research has indicated that the addition of Trichoderma can reduce the CF content of corn stalks by approximately 23.5% (Li et al., 2022), while fermentation with *A. niger* can increase the protein content by about 22% (Fan et al., 2022). The increase in protein content is particularly important in providing cost-effective feed, as access to affordable and high-quality feed is a crucial constraint in animal nutrition. Solid-state fermentation of DDGS not only reduces anti-nutritional factors but also increases protein content through bacterial proliferation. Despite the relatively long fermentation period, the increased CP content significantly enhances the competitiveness of DDGS as a high-protein feed.

Studies have demonstrated that feeding fermented DDGS significantly enhances the average daily gain of swine, reduces average daily feed intake and feed-to-weight ratio, and induces beneficial changes in the microbial flora of swine manure (Wang et al., 2017). In summary, solid-state fermentation of DDGS substantially reduces lignocellulosic content and eliminates the negative effects of anti-nutritional factors in the feed on monogastric animals. Concurrently, the increase in CP content improves the competitiveness of DDGS as a protein feed.

## 5. Conclusion

NSPs include cellulose, xylan, pectin, and smaller amounts of mannan and galactomannan. These complex carbohydrates can have detrimental effects on animal performance as animals lack the necessary digestive enzymes to break them down. Additionally, certain NSPs possess high viscosity due to their network structure, leading to the inhibition of nutrient digestion by adsorbing digestive enzymes. Therefore, it is crucial to identify microorganisms that possess the ability to extensively degrade NSPs. In this study, we conducted a systematic analysis of the potential NSPs-degrading enzymes present in the genome of *P. pabuli* E1. The genome of *P. pabuli* E1 encodes a diverse range of carbohydrate-degrading enzymes. By examining the distribution of carbohydrates, this study provides essential data that can be utilized for the development and application of degrading enzymes. *In vitro* enzyme activity assays were conducted, which demonstrated that *P. pabuli* E1 produces enzymes capable of effectively degrading NSPs. Furthermore, fermentation experiments using DDGS confirmed the practical applicability of *P. pabuli* E1 in eliminating non-starch polysaccharides. Given the presence of numerous potential NSPs-degrading enzymes in *P. pabuli* E1, this microorganism holds significant promise as a candidate for the development of highly efficient enzyme preparations.

## Data availability statement

The datasets presented in this study can be found in online repositories. The names of the repository/repositories and accession number(s) can be found at: https://www.ncbi.nlm.nih.gov/genbank/, MT322455.

## Author contributions

GL contributed to performing the experiments, analyzing data, and writing the initial draft. YY performed bioinformatics analysis of NSPs enzyme. BJ and ZZ were responsible for testing samples and collecting data. HZ and BM performed the instrument and revised the language of the article. The project fund management and final manuscript preparation were performed by YX, XL, and LW. All authors contributed to the article and approved the submitted version.

## Funding

This project was supported by the International (Regional) Cooperation and Exchange Program of the National Natural Science Foundation of China (grant no. 41861124004).

## Conflict of interest

The authors declare that the research was conducted in the absence of any commercial or financial relationships that could be construed as a potential conflict of interest.

## Publisher’s note

All claims expressed in this article are solely those of the authors and do not necessarily represent those of their affiliated organizations, or those of the publisher, the editors and the reviewers. Any product that may be evaluated in this article, or claim that may be made by its manufacturer, is not guaranteed or endorsed by the publisher.
