# Supplementary figures and images for: Feasibility insights into the application of Paenibacillus pabuli E1 in animal feed to eliminate non-starch polysaccharides
Source: Front Microbiol. 2023 Aug 7;14:1205767. doi: 10.3389/fmicb.2023.1205767 (PMC10440823; doi:10.3389/fmicb.2023.1205767)

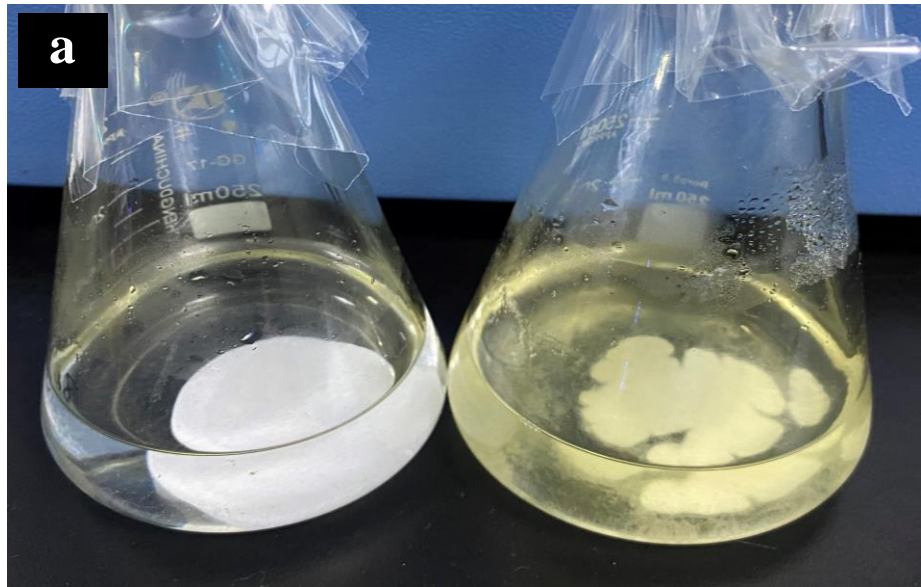

**control**

**supernatant**

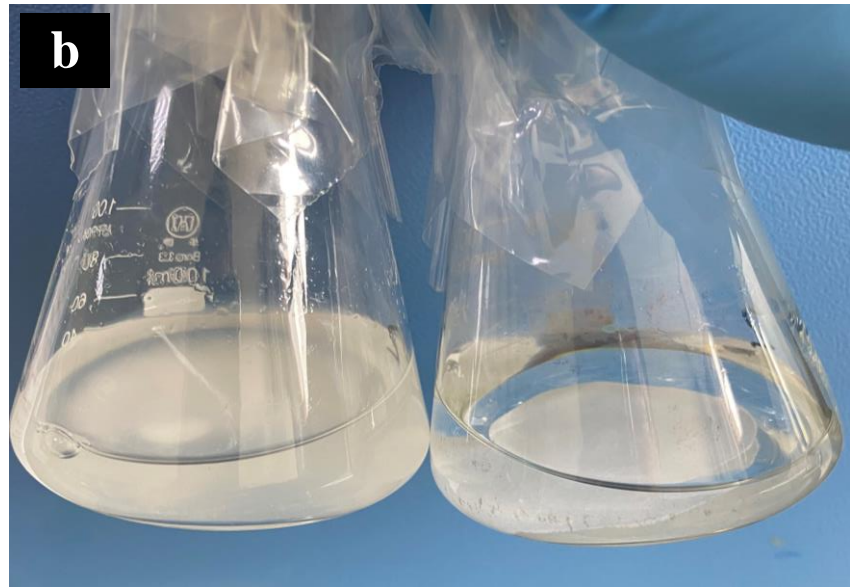

**cell insoluble fraction**

**control**

Supplement: Supplementary file 1 [file Image_1.pdf]
